# PB.40. What happens to the ductal carcinoma *in situ *in HER2-positive cancers treated with neoadjuvant chemotherapy and trastuzumab?

**DOI:** 10.1186/bcr3735

**Published:** 2014-11-03

**Authors:** R Millican-Slater, D Dodwell, K Horgan, M Mcmahon, B Dall, N Sharma

**Affiliations:** 1 Leeds Teaching Hospital NHS Trust, Leeds, UK

## Introduction

There are few data regarding the effect of neoadjuvant chemotherapy (NACT) and trastuzumab on any ductal carcinoma *in situ *(DCIS) associated with the HER2-positive invasive carcinoma. HER2-positive breast cancers are more likely to achieve a pathological complete response (defined as absence of invasive tumour in the final excision specimen allowing for the presence of DCIS). We review our data to see what happens to HER2-positive DCIS associated with HER2-positive invasive cancer treated with NACT.

## Methods

All cases that were HER2-positive from our local NACT database were identified from 2010 to 2012. The imaging features, core biopsy and final histology were documented

## Results

A total 41 of 150 patients that received NACT (anthracycline and taxane based) were HER2-positive and treated with trastuzumab. In total, 40/41 cases had surgery following neoadjuvant treatment, 24/41 (59%) cases had calcifications on mammography, and 21/41 (51%) obtained a pCR. Of the 24 cases with calcification, 14 (44%) had a pCR with 9/14 (29%) having residual DCIS. Seven of 24 cases had a WLE in which the calcifications on mammography were unchanged in extent or appearance. A total 17/41 were without calcification, seven (57%) had a pCR with 2/7 (62%) having residual DCIS that was noncalcified.

## Conclusion

pCR is higher in the HER2-positive group compared with the HER2-negative group (51% vs. 20%). In total, 48% achieved a pCR with no residual DCIS but 52% still had residual DCIS, suggesting that trastuzumab may be less effective in treating HER2-positive DCIS.

